# The Teaching of Hygiene and Temperance to School Children

**Published:** 1904-02-06

**Authors:** 


### THE TEACHING OF HYGIENE AND TEMPERANCE TO SCHOOL CHILDREN.

THE proposal to present to the authorities who are concerned with the function of education a petition embodying the opinion of the medical profession in favour of giving to children in primary and secondary schools elementary instruction in health subjects, including temperance, is one which will naturally command general sympathy. It can hardly be anticipated that any serious objection will be raised to the suggestion that the educational scheme shall include an attempt to introduce children to the truth that health of body depends in a large measure upon obedience to the rules and methods of natural law. To particular groups and sections school education, rightly or wrongly, is the opportunity for indoctrinating the children with certain ideas that may or may not be of importance. But it concerns the State that the training of school life shall issue in the production of citizens capable of contributing to the sum of national energy and fitted to play an active part in the world's work. Hence, when it is once allowed that such instruction as is now suggested is calculated to develop in the youthful mind an accurate appreciation of the value and importance of good health, it follows as an inevitable conclusion that it is the duty not less than the interest of the State to include such instruction in the compulsory curriculum.

There will be no hesitation in allowing that the medical profession has a special right to make its voice heard on this question. Necessarily a special responsibility is attached to those who from their training and experience are particularly fitted to suggest methods by which the health and vigour of the community may be promoted. So far from overstepping their province the organisers of the petition now in question have in our opinion shown themselves to be responsive to the claim of public duty and worthy of the support of all patriotic citizens. Recent investigations regarding the physical condition of children attending the public schools in the poorer quarters of the larger towns and of youths offering themselves for service in the army proclaim a state of matters which it is no exaggeration to term a national danger and thus the prayer of the petitioners appears at a peculiarly appropriate date.

Not less important than the principle for which the petitioners contend is the manner in which the suggested instruction should be carried out. It is obvious that the teaching of elementary physiology may lead, as is allowed, to the development of unwholesome tendencies, and lessons on "temperance" may easily be converted into examples of exaggeration and excess. Further, it is quite possible to present such teaching as a mere scholastic routine which will have no effect on the minds and lives of the scholars. Scientific details of bodily function or of "the nature and effects of alcohol" cannot be expected to influence the habits of children, and the true method is to be found not in the denunciation of certain practices, but in the exhibition of examples of virtue and moral worth. The complement to the prayer of the petition would be found in a series of lessons showing ancient and modern instances of the practice of temperance, self-control, physical righteousness, and "all that makes a man." It is not by mere formal knowledge, but by the stimulus of sympathetic example that children will be urged to high endeavour. To treat instruction in personal hygiene and temperance as a lesson in science which conveys warnings to the individual will have little or no value. Such instruction if it is to carry benefit to the individual and the nation must be part of an ethical code in which these and other virtues are presented in concrete examples that appeal to the generous heart of youth and prompt and strengthen the sense of duty as a noble and heroic obligation. For these ends neither scraps of scientific hygiene nor the recital of dramatic pathological penalties will furnish an adequate or abiding motive.

